# Recurrence of Basal Cell Carcinoma across Different Treatment Modalities: A Nationwide Study with 8 Years of Follow-up and Modelled Prediction

**DOI:** 10.2340/actadv.v106.adv-2025-0064

**Published:** 2026-07-09

**Authors:** Johan Sieborg, Emily Wenande, Alexander Egeberg, Henrik Sølvsten, Ulrikke Lei, Emilie Westerlin Kjeldsen, Merete Haedersdal

**Affiliations:** 1 Department of Dermatology, Copenhagen University Hospital – Bispebjerg and Frederiksberg, Copenhagen, Denmark; 2 Department of Clinical Medicine, Faculty of Health and Medical Sciences, University of Copenhagen, Copenhagen, Denmark; 3 Dermatology Centre North, Aalborg, Denmark; 4 Department of Dermatology and Allergy, Copenhagen University Hospital – Herlev and Gentofte, Copenhagen, Denmark

**Keywords:** Basal cell carcinoma, Epidemiology, Keratinocyte carcinoma, Nonmelanoma skin cancer, Machine learning, Recurrence, Treatment

## Abstract

Despite the rising incidence of basal cell carcinoma (BCC), few studies have compared treatment outcomes or predicted recurrences using nationwide real-world datasets. Hence, the objective was to investigate and compare recurrence rates of nodular BCC (nBCC) and superficial BCC (sBCC) following treatment with standard excision (SE), curettage, cryotherapy, photodynamic therapy and imiquimod. Additionally, we identified predictors of BCC 2-year recurrence using machine-learning. The study examined BCCs treated in state-funded, office-based dermatology practices registered in the Danish Skin Cancer Registry from 2014 to 2024. A total of 131,119 tumours were included, comprising 100,221 nBCCs and 30,898 sBCCs. For nBCC, the lowest rate of recurrence was observed for SE, with an 8-year cumulative recurrence rate (CRR) of 16.5% (95% confidence interval: 14.4–19.0%) and hazard ratio of 0.80 (0.76–0.84) compared with curettage. For sBCC, both SE and curettage had similarly low 8-year CRRs of 16.3% (12.7–20.8%) and 16.3% (15.2–17.5%), respectively, with no significant difference in hazard. The machine-learning model identified key predictors of BCC recurrence, including facial location, larger tumour size, having a high number of hospital-registered diagnoses and short distance to dermatological clinics. These findings highlight the impact of treatment selection and patient-specific factors in guiding dermatologists towards optimal BCC management.

**SIGNIFICANCE**

Basal cell carcinoma (BCC) is the most common skin cancer worldwide, with incidences increasing steadily over recent decades. This mounting burden on healthcare systems highlights the need for efficient treatment selection to reduce recurrence and optimise patient outcomes. Our results showed that for nodular BCC, recurrence rates were lowest for standard excision. For superficial BCC, standard excision and curettage had similarly low recurrence. A machine-learning analysis showed having a high number of hospital-registered diagnoses and short distance to dermatological clinics were predictors of 2-year BCC recurrence. Our findings may help dermatologists tailor treatment and follow-up strategies to improve BCC management.

Basal cell carcinoma (BCC) is the most common skin cancer worldwide, with incidence rates among individuals 65 or older projected to rise 140% by 2050 (1, 2). In Denmark**,** tumour-based incidence rates of BCC have more than doubled over the last decade, reaching 398 per 100,000 person-years in 2021–2022 (3). The increasing burden on healthcare systems underscores the need for judicious treatment selection to reduce recurrence and optimise patient outcomes. At present, no study exceeding 100,000 BCC tumours (4–7) has compared recurrence rates across different treatment modalities or developed recurrence prediction models using real-world data.

In clinical practice, BCCs are commonly classified as “easy-to-treat” (low-risk tumour) or “difficult-to-treat” (high-risk tumour) based on the histological subtype, tumour size, anatomical location and whether the tumour is recurrent. In addition to risk classification, treatment choice is further guided by non-tumour-related considerations such as patient profile, preference and cosmetic outcome (8). For the most common BCC subtypes, nodular BCC (nBCC) and superficial BCC (sBCC), treatment options for low-risk tumours include standard excision (SE), destructive treatments (curettage, electrodessication and cryotherapy) and topical treatments (imiquimod, 5-fluorouracil or photodynamic therapy [PDT]) (8). For high-risk BCC, other treatment options are often selected, including surgery with margin control, radiotherapy and more rarely, Hedgehog inhibitors.

Recurrence rates vary widely across treatment modalities partly reflecting treatment selection based on anatomical location, tumour size, and histological subtype. For SE, studies report 5-year cumulative recurrence rates (CRRs) of 0.8–8.4% for completely excised tumours, but 30–41% for incompletely excised tumours (9–11). Beyond SE, other common BCC treatments show even greater variation, with 5-year CRRs of 3.3–22.7% for curettage and electrodessication (5, 12, 13), 1–13% for cryotherapy (14–17), and 6–45% for PDT (18, 19), and a 3-year CRR of 14.3–28.4% for sBCC treated with imiquimod (20). Importantly though, few studies compare different treatments while accounting for tumour size, anatomical location and histological subtype, and long-term CRRs remain largely unknown. In addition to treatment modality, other non-tumour-related factors such as individual healthcare utilization and other patient-related factors may influence CRRs in real-world clinical settings. Models predicting recurrence in BCC tumours may not only help aid in early identification of recurrent tumours but may also expand our current knowledge regarding the relative impact of non-tumour-related factors on treatment outcomes. For example, excellent machine learning (ML) models have been developed to predict early recurrence of breast cancer (21–23). In dermatology, their potential for predicting recurrent BCC has yet to be explored. Such models can capture complex correlations, offering deeper insights into recurrence patterns. The aim of this study was to compare BCC recurrences following treatment with curettage, SE, imiquimod, PDT or cryotherapy using real-world registry data from all Danish dermatological practices. Additionally, ML models based on a boosted decision tree were developed to predict factors impacting 2-year recurrence of nBCC and sBCC.

## METHODS

### Data sources

Data were extracted from the Danish Skin Cancer Registry, which is a part of the Danish Clinical Quality Program for National Clinical Registries and contains data from office-based dermatological practices participating in the state-funded public healthcare system (24). The registry has records of treatment choice, recurrent tumours and tumour/patient characteristics including BCC histological subtype, lateral tumour size (diameter), Fitzpatrick skin type and anatomical location. In addition to the Danish Skin Cancer Registry, the Civil Registration System, the Income Statistics Register, the National Patient Register and the Danish Education Register (25) were used to extract civil information about age, sex, individual disposable income, residential location, hospital-registered diagnoses and educational attainment. Study population and inclusion/exclusion criteria.

The study population consisted of all primary and recurrent BCC tumours treated in office-based dermatological practices recorded in the Danish Skin Cancer Registry between January 2014 and December 2024. BCC tumours referred to specialties of plastic surgery or oncology were excluded. Tumours with no follow-up visit were excluded. Both clinically and histologically diagnosed tumours were included, where histologic diagnosis/verification overruled the clinical diagnosis when available. In cases where no BCC subtype was specified by histology, the clinically diagnosed subtype was used.

### Outcomes and variable definitions

The primary outcome was recurrence of nBCC and sBCC following treatment with curettage, SE, imiquimod, PDT, cryotherapy and 5-fluorouracil. Recurrence was assessed using CRRs, hazard ratios (HRs) and ML recurrence prediction model. CRRs and HRs was determined using time to recurrence as end point, whereas the ML prediction model used recurrence within 2 years after treatment as its end point.

Recurrence was recorded by a practicing dermatologist and was defined as a recurrent tumour identified either during a control visit or in connection with other visits to the office-based dermatological clinic. Follow-up time was defined as the time from treatment to diagnosis of a recurrent tumour or to date of the last registered follow-up visit. Throughout the study period, 2 guidelines were in use (2008–2019 (26) and 2019–2026(27)), with slightly different follow-up recommendations. The 2008–2019 guideline recommended 2 follow-up visits for high-risk BCCs, 1 at 3–6 months and another at 12 months, while no clear follow-up recommendations were provided for low-risk BCCs. The 2019–2026 guideline recommended 2 follow-up visits for all BCCs, at 3–6 months and at 12 months. Treatments in the study included curettage, SE, imiquimod, PDT, cryotherapy and 5-fluorouracil. Curettage was defined as all variants of curettage including double curettage with electrodesiccation (85.0% of all curettage), single curettage with electrodesiccation (5.8%), curettage with cryotherapy (3.8%) or curettage with silver nitrate (2.9%) or unspecified curettage (2.5%). PDT was conventional PDT or daylight PDT using methyl aminolevulinate or 5-aminolevulinic as photosensitising agent. It was not possible to distinguish between the 2 PDT methods, as both are recorded as PDT. A 4 mm safety margin cut-off was chosen for excision, as this is the recommended margin for low-risk BCCs according to National Comprehensive Cancer Network (2024) (28) and British Association of Dermatologists (2021) (29) BCC guidelines.

Treating dermatologists noted the anatomical location with *International Statistical Classification of Diseases and Related Health Problems* (ICD) 10 code. Anatomical location was categorized into unspecified face (C443, D043), lip (C440, D040), eye (C441, D041), ear (C442, D042), neck or scalp (C444, D044), upper extremity (C446, D046), trunk (C445, D045) and lower extremity (C447, D047). Tumour size is defined as the lateral tumour size and categorized into 5 groups as <5 mm, 5–10 mm, 10–15 mm, 16–20 mm, >20 mm or unknown (8).

Number of hospital-registered diagnoses was captured as the number of ICD-10 diagnosis registered in a hospital setting for a given patient prior to BCC treatment. Comorbid skin disease was defined as the presence of at least one hospital-registered ICD-10 diagnosis of psoriasis (L40), atopic dermatitis (L40, L208D), other eczema (L308H, L858J, L249, L240A, L235H, L308I), hidradenitis suppurativa (L732), rosacea (L71), urticaria (L50, L282A, L563) or acne (L70). Educational attainment was categorized into higher education (bachelor or higher education), basic education (elementary-school, high-school), vocational education or unknown. Distance to dermatological clinic was determined on a municipality (i.e. district) level by measuring the distance from the middle of the residential municipality (30) to the nearest dermatological clinic in kilometres (km).

### Statistics

CRRs were estimated with Kaplan–Meier analysis, and HRs were estimated with Cox proportional hazards regression. Variables were considered significant within a 95% confidence interval (CI). Multivariable adjustment in Cox-regression included age, sex, anatomical location, tumour size and Fitzpatrick skin type. Scaled Schoenfeld residuals were analysed to ensure the Cox proportional assumption was fulfilled. There was no suspicion of nonproportionality except for PDT treatment for nBCC. To maintain the Cox proportional hazards assumption, analyses were stratified into 3 calendar-year groups for PDT treatment for nBCC: 2014–2018, 2019–2020 and 2021–2024. Cox-regression analysis of 5-fluorouracil treatment was limited to sBCC tumours, due to the small number of nBCCs treated with this modality. Curettage was selected as the reference group in the Cox-regression, as it was the most frequently used treatment method for both nBCC and sBCC. An additional analysis was prediction of factors impacting 2-year recurrence using an ML model. For the ML prediction model, all patients treated between 2023 and 2024 were excluded due to inadequate follow-up duration (i.e. less than 2 years since treatment at time of analysis). To construct an ML prediction model, boosted decision tree was applied. Randomized search and nested cross-validation were applied to optimize the model and reduce overfitting. Further, Shapley (SHAP) values were calculated to estimate predictor importance. The ML model used the following variables to predict recurrent sBCC or nBCC tumour: treatment method, histological subtype, anatomical location, tumour size, individual disposable income, distance to dermatological clinic, educational attainment, sex, age, number of hospital-registered diagnoses and presence of comorbid skin diseases. Receiver operator curve (ROC) analysis (area under the curve [AUC], accuracy) were used to evaluate the results of the ML models. All statistics were performed in Python 3.7.4 (Python Software Foundation).

## RESULTS

### Patient and tumour characteristics

The study included 188,663 BCC tumours, of which 57,544 (30.5%) were excluded due to missing follow-up data. The remaining 131,119 BCC tumours consisted of 100,221 (76.4%) nBCC tumours and 30,898 (23.6%) sBCC tumours (Table I). The average number of BCCs per patient in the study period was 1.95. Overall, 94% of BCCs were histologically verified, while the remaining 6% were based on clinical assessment by specialists in dermatology. nBCC tumours were treated at a median patient age of 73.3 years, while sBCC tumours were treated at a slightly younger median age of 70.7 years. The median follow-up time was 1.0 year (IQT: 0.6–2.1 years) for nBCCs and 1.3 (IQT 0.7–3.2 years) for sBCCs. A total of 25,056 nBCCs and 7,725 sBCCs had follow-up periods exceeding the upper quartile (2.1 and 3.2 years, respectively). Follow-up of more than 5 years was available for 9,037 nBCCs and 4,452 sBCCs.

**Table I.** Baseline tumour and patient characteristics of nodular (nBCC) and superficial basal cell carcinoma (sBCC)

	All	nBCC	sBCC
Total number of patients, *n*	67,071	56,375	18,224
Total number of patients treated between 2014 and 2022, n*	57,394	47,580	15,970
Total number of tumours, *n*	131,119	100,221	30,898
Men, *n* (%)	31,760 (47.4)	27,376 (48.6)	8,083 (44.4)
Age at treatment, median (IQT)	72.7 (64.1,78.9)	73.3 (65.0,79.3)	70.7 (61.1,77.3)
Distance to nearest clinic (km), median (IQT)*	6.9 (2.5,12.8)	6.9 (2.3,12.5)	7.6 (3.3,13.6)
Number of hospital-registered diagnoses, median (IQT)*	5.0 (0.0,18.0)	6.0 (0.0,19.0)	3.0 (0.0,15.0)
Annual disposable income Euro, median (IQT)*	55,668 (38880,86083)	55,061 (38804,84808)	57,524 (39189,89887)
Educational attainment, *n* (%)*			
Higher education	18,810 (32.8)	15,321 (32.2)	5,610 (35.1)
Basic education	14,891 (25.9)	12,473 (26.2)	3,875 (24.3)
Vocational education	22,930 (40.0)	19,163 (40.3)	6,255 (39.2)
Unknown	763 (1.3)	623 (1.3)	230 (1.4)
Treatment			
Curettage	104,726 (79.9)	85,489 (85.3)	19,237 (62.3)
Excision - Surgical margin <4 mm	11,872 (9.1)	9,990 (10.0)	1,882 (6.1)
Excision - Surgical margin ≥4 mm	1,553 (1.2)	1,276 (1.3)	277 (0.9)
Photodynamic therapy	5,097 (3.9)	2,352 (2.3)	2,745 (8.9)
Imiquimod	3,705 (2.8)	547 (0.5)	3,158 (10.2)
Cryotherapy	3,982 (3.0)	540 (0.5)	3,442 (11.1)
5-Fluorouracil	184 (0.1)	27 (0.0)	157 (0.5)
Tumour size, *n* (%)			
< 5 mm	41,695 (31.8)	32,646 (32.6)	9,049 (29.3)
6–10 mm	56,944 (43.4)	44,357 (44.3)	12,587 (40.7)
11–15 mm	15,739 (12.0)	11,535 (11.5)	4,204 (13.6)
16–20 mm	4,895 (3.7)	3,382 (3.4)	1,513 (4.9)
> 20 mm	2,737 (2.1)	1,633 (1.6)	1,104 (3.6)
Unknown	9,109 (6.9)	6,668 (6.7)	2,441 (7.9)
Anatomical locations, *n* (%)			
Face (Unspecified)	38,581 (29.4)	32,916 (32.8)	5,665 (18.3)
Scalp or neck	10,193 (7.8)	8,658 (8.6)	1,535 (5.0)
Eye	588 (0.4)	506 (0.5)	82 (0.3)
Ear	2,028 (1.5)	1,754 (1.8)	274 (0.9)
Lip	412 (0.3)	330 (0.3)	82 (0.3)
Upper extremity	14,846 (11.3)	10,640 (10.6)	4,206 (13.6)
Trunk	53,072 (40.5)	37,846 (37.8)	15,226 (49.3)
Lower extremity	11,399 (8.7)	7,571 (7.6)	3,828 (12.4)
Fitzpatrick skin type, *n* (%)			
Type 1	4,063 (6.1)	3,461 (6.1)	1,145 (6.3)
Type 2	45,634 (68.0)	38,732 (68.7)	11,146 (61.2)
Type 3	9,171 (13.7)	7,127 (12.6)	2,993 (16.4)
Type 4, 5, or 6	107 (0.2)	86 (0.2)	30 (0.2)
Unknown	8,096 (12.1)	6,969 (12.4)	2,910 (16.0)

*Variables for the machine-learning algorithm are presented exclusively for BCC patients treated between 2014 and 2022.

DKK was converted to Euro with an exchange rate of 7.44 to 1**.**

IQT: interquartile; PDT: photodynamic therapy.

The most common anatomical location for nBCCs was the head and neck area (face+scalp and neck, 41.4%), while for sBCC, the most common location was the trunk (49.3%) (Table I). Tumour size was smaller for nBCC compared to sBCCs, with 76.9% of nBCCs and 70.0% of sBCCs measuring less than 10 mm. Curettage was the most frequently used treatment method for both nBCC and sBCC, accounting for 85.3% and 62.3%, respectively (Table I).

### Cumulative recurrence rates

**Fig. 1.**
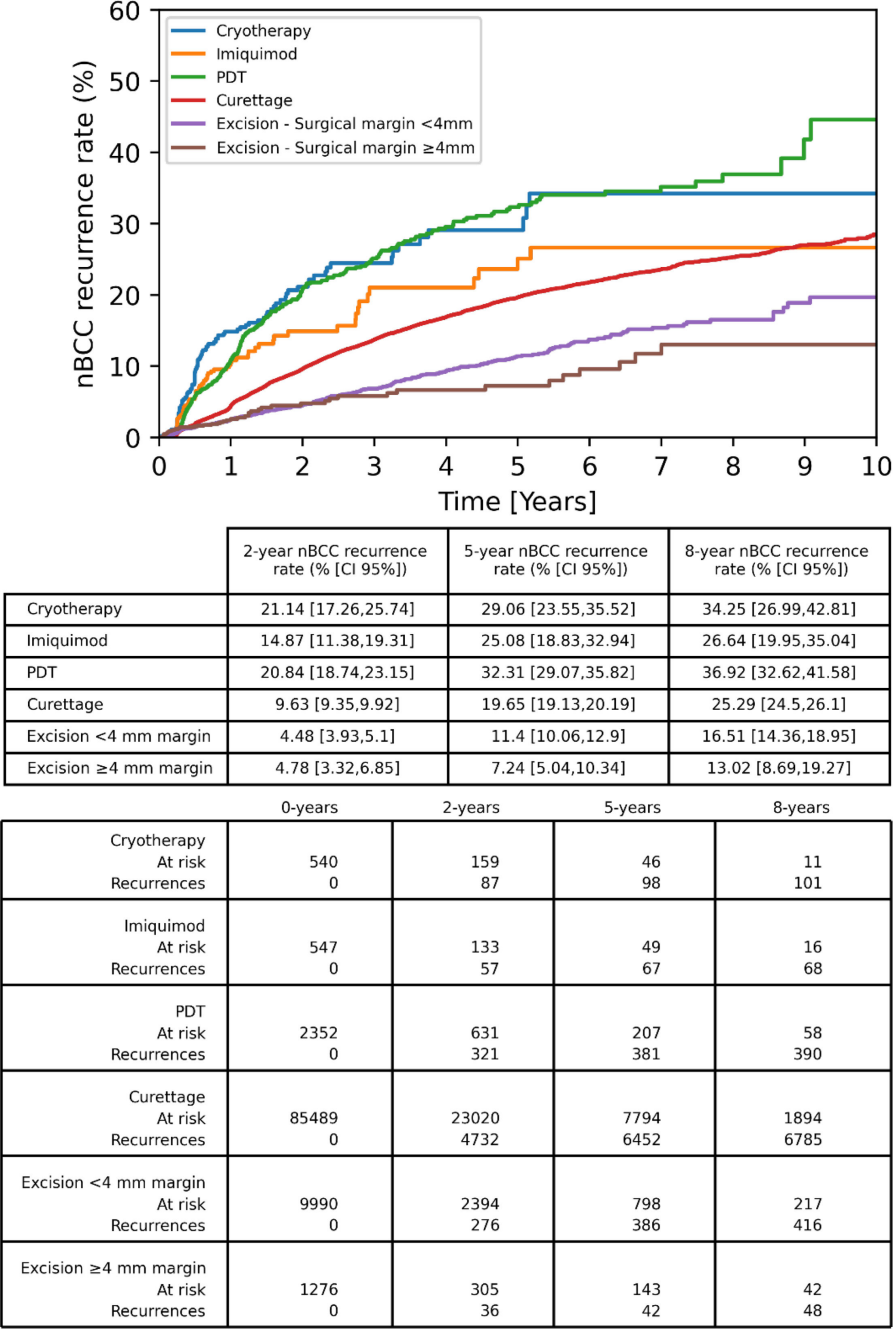
Cumulative recurrence rate with corresponding at risk population estimated with Kaplan–Meier analysis and stratified by treatment type for nodular basal cell carcinoma (nBCC). PDT: photodynamic therapy.

**Fig. 2.**
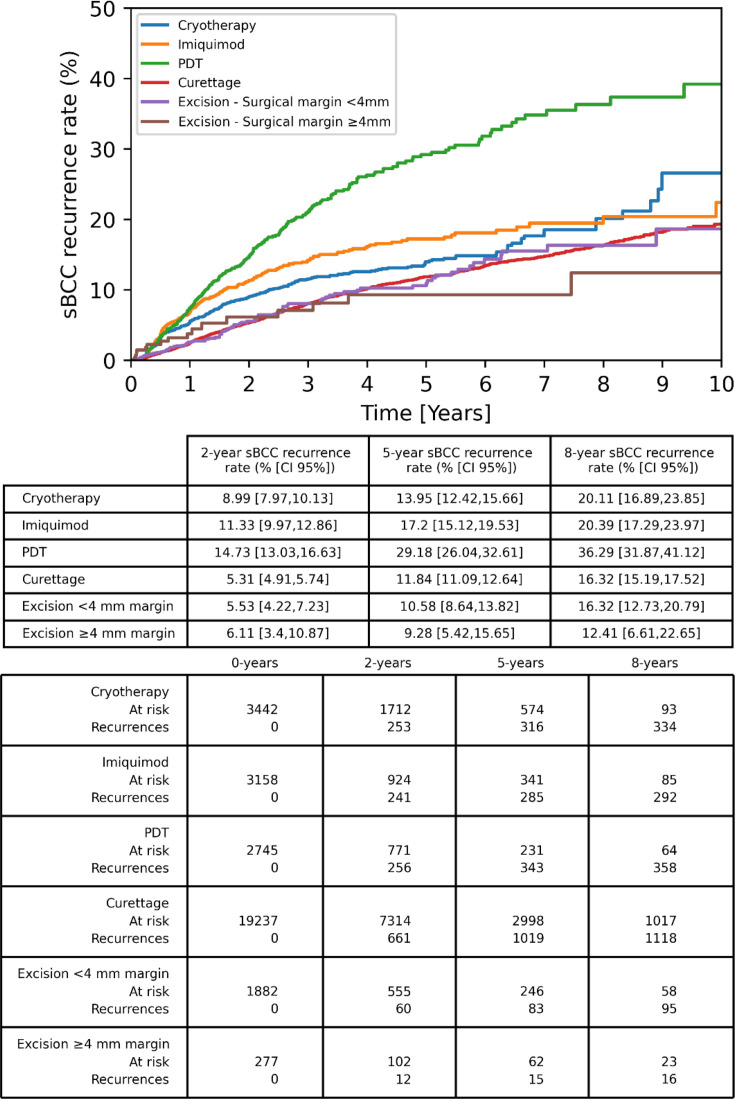
Cumulative recurrence rate with corresponding at risk population estimated with Kaplan–Meier analysis and stratified by treatment type for superficial basal cell carcinoma (sBCC). PDT: photodynamic therapy

Cumulative recurrence rates increased throughout the 10 years of follow-up for both nBCC and sBCC, with specific rates specified at years 2, 5 and 8 (Figs 1 and 2). For nBCC, the highest rate of recurrence as measured by CRR was observed for PDT, with an 8-year CRR of 36.9%. The lowest CRR was noted for SE using a ≥4 mm margin, with an 8-year CRR of 13.0%, followed by SE with a margin <4 mm (16.5%) and curettage (25.3%). Few nBCC treated with cryotherapy or imiquimod had follow-up times longer than 3 years; however, the 2-year CRRs of 21.1% for cryotherapy and 14.9% for imiquimod were higher than for nBCC treated with curettage (2-year CRR: 9.6%).

For sBCC, the highest recurrence rates were observed for PDT, with an 8 year CRR of 36.3% (Fig. 2). Lower sBCC recurrences were observed for SE margin<4/≥4 mm (8 year CRR of 16.3% and 12.4%, respectively) and curettage (16.3%). Compared with nBCC, sBCC tumours treated with cryotherapy and imiquimod recurred at lower rates, displaying 2-year CRRs of 9.0% and 11.3%, respectively.

CRRs exhibited slower recurrence rates beyond the 5-year mark regardless of treatment and tumour subtype, as illustrated in Figs 1 and 2.

### Hazard ratios for recurrence

**Fig. 3.**
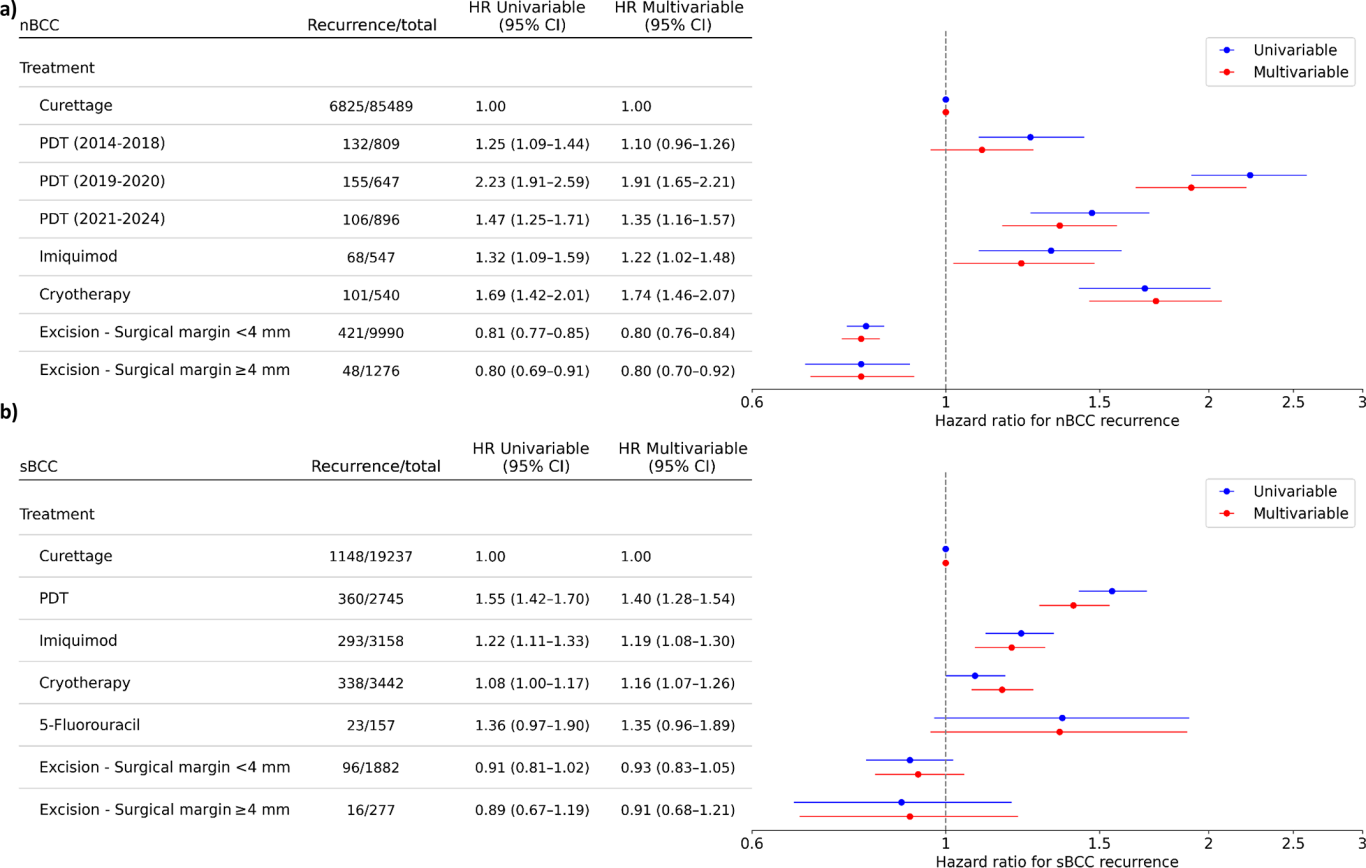
Hazard ratios for recurrent tumour according to treatment method in a) nodular basal cell carcinoma (nBCC), b) superficial basal cell carcinoma (sBCC). Multivariable adjustment from Cox-regression included age, sex, tumour size, anatomical location, and Fitzpatrick skin type. HR: hazard-ratio; PDT: photodynamic therapy

For both nBCC and sBCC, similar patterns in HRs of recurrence were observed for the different treatment methods (Fig. 3). SE showed 20% lower hazard of recurrence for nBCC compared to curettage as reference, with HRs of 0.80 (0.76–0.84) for<4 mm margin and 0.80 (0.70–0.92) for≥4 mm margins. Conversely, increased HRs of recurrence were observed for nBCCs treated with PDT with HRs ranging from 1.10 to 1.91 depending on calendar year, imiquimod with a HR of 1.22 (1.02–1.48) and cryotherapy with a HR of 1.74 (1.46–2.07).

For sBCC, no significant differences in HRs were observed for SE with<4/≥4 mm margin and 5-fluorouracil compared with curettage. Increased HRs were observed for sBCCs treated with PDT with a HR of 1.40 (1.28–1.54), imiquimod 1.19 (1.08–1.30), and cryotherapy 1.16 (1.07–1.26).

### Prediction of 2-year BCC recurrence

**Fig. 4.**
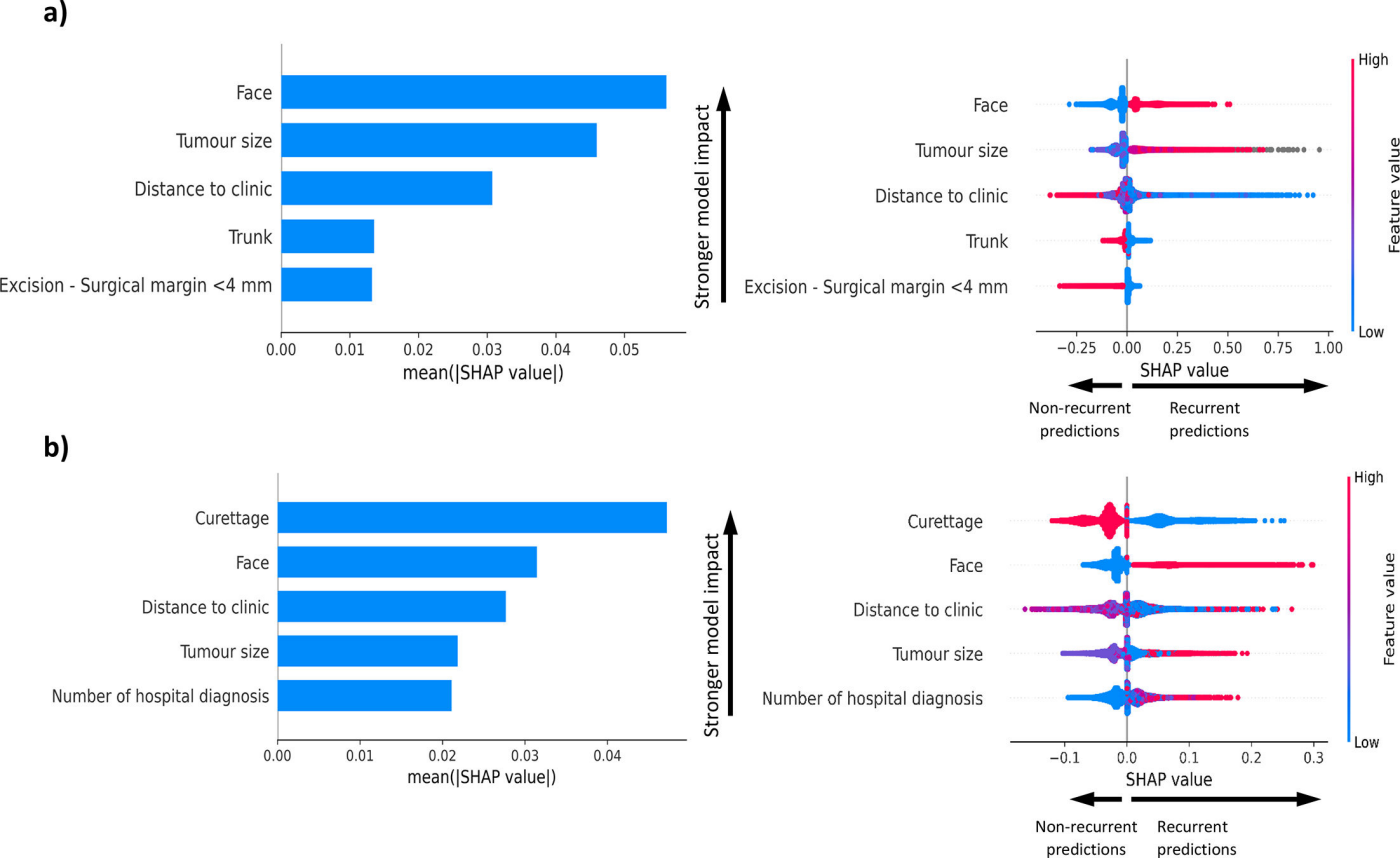
Predictors of 2-year BCC recurrence based on a machine learning model. The left panel show the predictors absolute mean Shapley (SHAP) value indicating the importance of each variable in the machine learning model. The right panels show SHAP values for the model using a boosted decision tree predicting 2-year BCC recurrence. **a**) Nodular basal cell carcinoma (accuracy: 0.60, ROC AUC: 0.68). b) Superficial basal cell carcinoma (accuracy: 0.66, ROC AUC: 0.71).

In general, the top 5 predictors of both nBCC and sBCC recurrence within 2 years from treatment included facial anatomical location, larger tumour size and a short distance to nearest dermatological clinic (Fig. 4). However, predictors of nonrecurrence differed between the 2 BCC subtypes. For nBCC, treated with excision and truncal location were important factors for nonrecurrence. For sBCC, treated with curettage and having a low number of hospital-registered diagnoses were important factors for nonrecurrence. Both ML models predicting nBCC and sBCC recurrence within 2 years after treatment demonstrated moderate discriminatory ability, achieving ROC AUC scores of 0.68 and 0.71, indicating a fair capacity to distinguish between tumours that did and did not recur, with an accuracy of 59.98% for nBCC and 66.15% for sBCC.

## DISCUSSION

This national registry-based study analysed just over 130,000 BCC treatments from office-based dermatological practices to assess the association between different treatments and BCC recurrence. Our findings showed SE was associated with the lowest hazard of nBCC recurrence, while for sBCC, no significant difference in recurrence hazard was detected between SE and curettage. In contrast, treatment with PDT, cryotherapy and imiquimod were linked to an increased hazard of recurrence for both tumour subtypes. Key predictors of recurrence identified by an ML model included facial location, larger tumour size, treatment with curettage or excision using a margin less than <4 mm, having a high number of hospital-registered diagnoses and close proximity to a dermatological practice. As a whole, these results highlight the importance of both tumour- and non-tumour-related factors in predicting BCC recurrence. Our real-world study underscores the relevance of considering these factors when selecting treatment and demonstrates the potential of prediction tools to better tailor follow-up strategies for more personalised BCC management.

Our findings align with previous studies on BCC recurrence rates. The 5-year CRRs for both nBCC and sBCC observed in this study fit within the previously reported ranges. Prior studies reported 5-year CRRs of 3–23% for curettage (5), 1–8% (complete margins) and 30–41% (incomplete margins) for SE (9–11), 6–45% for PDT (18, 19), 1–13% for cryotherapy (14–17), and 14–28% (3-year recurrence rate) for sBCC treated with imiquimod (20). Comparable to our findings for nBCC, other BCC studies have indicated that SE outperforms curettage combined with cryotherapy (31), PDT (18, 32) and imiquimod (33). However, in contrast to our results for sBCC, similar recurrence rates have been reported between SE and PDT (34); curettage and cryotherapy (35). This difference may reflect differences in dermatology training, treatment procedure and technique, tumour size and anatomical location, or selection bias in treatment choices. Our findings should be interpreted in the context of tumour characteristics and anatomical location. Although nodular and superficial BCC are generally histologically low-risk subtypes, many occur on the face, including the H-zone, where recurrence risk is higher. This highlights that direct comparisons of cumulative recurrence rates may be misleading when tumour characteristics, particularly location, differ. In our analyses, facial location emerged as an important predictor of recurrence, but accounting for anatomical location in the Cox-analyses did not alter the overall treatment-specific recurrence patterns. The importance of anatomical location as a predictor of 2-year recurrence likely reflects a combination of its classification as a high-risk anatomical area and its higher visual detectability. Specifically, dermatologists may prioritize cosmetic outcomes during primary treatment of facial BCCs, while the area’s visibility facilitates early detection of any recurrence.

As the mounting BCC burden on the healthcare system continues to grow, appropriate treatment selection becomes increasingly important. Our findings for sBCC, which showed similar hazard for recurrence and 8-year CRR between curettage and SE (curettage: 16.3%, SE with <4 mm margin: 16.3%), may support the use of curettage for sBCC – a less resource-intensive treatment compared to SE. The curettage group included multiple variants, which may have a minor influence on the reported recurrence rate depending on the variant. No significant difference was observed between 5-fluorouracil and curettage or SE for sBCC, likely due to the small number of sBCCs treated with 5-fluorouracil resulting in wide CIs. Therefore, the results for 5-fluorouracil should be interpreted with caution. Additionally, selecting treatments associated with higher recurrence rates and hazard for recurrence may be difficult to justify in the context of a rising BCC burden. This includes PDT, for which our results showed a 40–91% increased hazard for recurrence, or cryotherapy and imiquimod for nBCC, with a 22% and 74% increased hazard, respectively, compared to curettage or SE. The association between year of PDT administration and recurrence in nBCC is likely multifactorial, and the exact reason remains unclear. Following the publication of Danish BCC guidelines in 2019, which endorsed PDT for low-risk BCC, the use of PDT increased. As PDT is an operator-dependent treatment, this broader uptake may partly explain the observed associations.

Long-term data on BCC recurrence rates beyond 5 years remain scarce. A 10-year follow-up study on SE and Mohs micrographic surgery found recurrence rates for primary tumours continued to increase at similar rates after the first 5 years (9). Similarly, our Kaplan–Meier analysis revealed a continued increase beyond 5 years after treatment, albeit at a slower rate. Our results, which were based on long follow-up times, may be useful for planning post-treatment monitoring, given that we show continued tumour recurrence even 10 years after treatment.

ML is fast becoming a commonly used tool in the medical field and has successfully been used to predict early recurrence of breast cancer (21–23) and oesophageal adenocarcinoma (36). The tool is not only useful to predict early recurrence but can also reveal patterns associated with recurrent tumours. Our ML models achieved decent ROC AUCs of 0.68 and 0.71 for nBCC and sBCC, respectively, indicating ability to discriminate between 2-year recurrent and 2-year nonrecurrent BCC tumours. The models identified well-established recurrence risk factors, such as larger tumour size and anatomical location on the face. Interestingly, treatment selection had a lower (for nBCC) or similar (for sBCC) impact on recurrence than other non-tumour-related variables, including a high number of hospital-registered diagnoses and a short distance to dermatological clinics. That only curettage and excision with <4 mm margins are among the top 5 predictors may reflect weaker associations of other treatment modalities with recurrence but may also be due to complex correlations between multiple covariates. The strong impact of having many hospital-registered diagnoses on the model suggests that these patients may either have a higher risk of recurrence or utilise/access the healthcare system more frequently, leading to higher detection rates. Furthermore, many hospital-registered diagnoses are presumably correlated with additional variables not included in the model, such as a higher comorbidity burden and increased medication use, which may also influence follow-up regimens. The finding that a short distance to the nearest dermatological clinic predicts recurrence could correspondingly indicate that patients residing at longer distances to dermatological clinics encounter great barriers to attending follow-up visits. Dermatologists should be mindful of these factors’ influence on the individual patient journey. Refining follow-up strategies by taking both tumour- and non-tumour-related factors into consideration could ensure more appropriate treatment, a more targeted and tailored follow-up plan, and in some cases, lower recurrence rates and improved patient outcomes.

In this real-world dataset, the main limitation of the study was the exclusion of one third of BCCs due to missing follow-up data. This could introduce bias, as patients without visible recurrence may be less likely to attend follow-up visits, potentially leading to overestimation of recurrence rates. Further, surgically treated tumours may be subject to closer clinical monitoring than less invasive treatments, leading to some detection and attrition bias. Moreover, treatment selection is influenced by tumour characteristics, patient profile and cosmetic considerations, which may therefore bias outcomes of different treatment modalities. This is reflected in the larger proportion of sBCCs receiving topical treatment compared with nBCCs, likely reflecting the superficial nature of this subtype. As the Danish Skin Cancer Registry captures data from office-based dermatological practices only, BCCs managed solely in primary care are not recorded, though this is considered a minor limitation as general practitioners rarely treat BCCs in Denmark. Additionally, some high-risk tumours may be referred to other departments, skewing the distribution of low- and high-risk tumours. To address this, we adjusted for key confounders in multivariable Cox-regression analyses. Nevertheless, the possibility of residual confounding cannot be entirely ruled out. It is worth noting that treating dermatologists could have a varying tolerance for recurrence depending on the clinical scenario. Besides minimizing BCC recurrence, other considerations such as cosmetic considerations, patient profile and preference may affect treatment choice and may feature more prominently in real-world data than RCT and clinical studies. Finally, although predictions models were able to discriminate between 2-year recurrent and 2-year nonrecurrent BCC tumours they might be further improved by including tumour images, histological tumour depth/thickness and specifically for SE, complete or incomplete margin status. The study has multiple strengths, including analysis of a nationwide real-world dataset that is several times larger than previous studies, with a follow up time up to 10 years. Previous CRR studies often included only single-centre studies from clinical trials or RCTs, with relatively small sample sizes not exceeding 4,000 tumours (4–7, 34, 37). Moreover, our study differentiated between the 2 most common BCC subtypes based on both histological and clinical diagnosis, an aspect known to influence recurrence rates notably. Additionally, this study compared multiple treatment options in a similar setting, allowing for fairer comparison between treatments as they are provided in a real-world dermatological practice setting. Another notable strength was the ability to adjust for tumour size and anatomical location across different treatment modalities, reducing treatment selection bias. Consequently, these findings should be applicable to other similar populations, although differences are expected due to different training regimens and treatment guidelines between countries (e.g. more frequent use of curettage in this Danish population).

In conclusion, our findings highlight substantial differences in recurrence risk across BCC treatment modalities. Nodular BCC treated with SE had the lowest recurrence, while both SE and curettage demonstrated low sBCC recurrence. PDT had the highest recurrence for both subtypes, with cryotherapy having a similarly high recurrence for nBCC specifically. For imiquimod, an intermediate recurrence rate between PDT and SE was noted for both subtypes. Moreover, the study identified both tumour- and non-tumour-related factors as important predictors of 2-year BCC recurrence. In addition to guiding treatment selection, these findings highlight a role for prediction tools to tailor follow-up strategies based on patient and tumour-related factors, ultimately improving BCC management.

## Data Availability

The data underlying this study cannot be shared publicly as Danish law does not allow transfer of these data.
